# A multigene typing system for human adenoviruses reveals a new genotype in a collection of Swedish clinical isolates

**DOI:** 10.1371/journal.pone.0209038

**Published:** 2018-12-14

**Authors:** Győző László Kaján, Agnieszka Lipiec, Dániel Bartha, Annika Allard, Niklas Arnberg

**Affiliations:** 1 Department of Clinical Microbiology, Virology, and Laboratory for Molecular Infection Medicine Sweden (MIMS), Umeå University, Umeå, Sweden; 2 Institute for Veterinary Medical Research, Centre for Agricultural Research, Hungarian Academy of Sciences, Budapest, Hungary; Sechenov First Medical University, RUSSIAN FEDERATION

## Abstract

Human adenoviruses (HAdVs) are common pathogens that can cause respiratory, gastrointestinal, urogenital, and ocular infections. They are divided into seven species containing 85 genotypes. Straightforward typing systems might help epidemiological investigations. As homologous recombination frequently shapes the evolution of HAdVs, information on a single gene is seldom sufficient to allow accurate and precise typing, and complete genome-based methods are recommended. Even so, complete genome analyses are not always easy to perform for practical reasons, and in such cases a multigene system can provide considerably more information about the strain under investigation than single-gene-based methods. Here we present a rapid, generic, multigene typing system for HAdVs based on three main deterministic regions of these viruses. Three PCR systems were used to amplify the genes encoding the DNA polymerase, the penton base hypervariable Arg-Gly-Asp-containing loop, and the hexon loop 1 (hypervariable region 1–6). Using this system, we typed 281 clinical isolates, detected members of six out of seven HAdV species (*Human mastadenovirus A*–*F*), and could also detect not only divergent strains of established types but also a new recombinant strain with a previously unpublished combination of adenovirus genomes. This strain was accepted by the Human Adenovirus Working Group as a novel genotype: HAdV-86. Seven strains that could not be typed with sufficient accuracy were also investigated using a PCR based on part of the fiber gene. By analysis of corresponding sequences of the 86 known HAdV genotypes, we determined that the proposed typing system should be able to distinguish all non-recombinant types, and with additional fiber information, all known HAdV genotypes.

## Introduction

The seven species of human adenovirus (*Human mastadenovirus A*–*G*; HAdV-A to HAdV-G) are divided into types, originally called serotypes. Virus strains were traditionally classified into serotypes on the basis of serum neutralization tests [1, 2]. These approaches are rather tedious and time consuming, and require numerous reference strains and antisera. Because of this, the classification of adenoviruses currently relies on molecular typing methods [3–6].

New or rare HAdV types may play a crucial role in industrial applications, as many known/common human adenovirus types are already patented, or pre-existing immunity limits their use in vector development. The most common HAdV-5 based vectors have been associated with liver- and innate immunity-associated toxicity [7]. Even so, adenovirus-based vectors have been developed for the treatment of both cancer and cardiovascular diseases, and for prevention of infectious diseases. Vector engineering is a very active and evolving field in virology [8–12].

To find possible new vector candidates (e.g. new and/or rare human adenovirus types), we have used molecular methods to type 281 human adenovirus strains that were isolated from patients in Sweden between 1978 and 2010. To ensure that we would not miss discovering any new recombinant genotypes, we used a multigene approach.

The external surfaces of the viral major capsid proteins―the hexon, the penton base, and the fiber [13]―contribute to antigenicity [14–17]; but the hexon loop 1 function as the major antigenic determinant that is mostly responsible for serotyping results [18]. The genes of these three proteins are therefore used in the description and characterization of new recombinant HAdV strains [19], so these would also be the best choice for multiple gene-based typing of HAdV strains. Apart from using the hexon gene and the penton base gene, a fiber-targeted PCR system was also tested using seven clinical isolates [20]. With six primer pairs, this multiplex PCR had been developed for species HAdV-A–F, but unfortunately it gave a low positivity rate (50%), and was therefore discarded from the set of PCR systems used. Also, since information from the DNA polymerase gene is required to classify adenoviruses into species [1], we decided to use the hexon gene, the penton base gene, and the polymerase gene.

## Materials and methods

### Origin of strains

The 281 HAdV clinical isolates analyzed were from Sweden: from Skåne University Hospital, Lund (n = 126) and from Norrland University Hospital, Umeå (n = 155). The strains had originated from diverse clinical conditions, and had been isolated on A549, Vero, Madin-Darby canine kidney, green monkey kidney or MA-104 cells. The original isolates were investigated without any further propagation.

### DNA extraction

DNA was extracted from 200 μL of isolate using a Qiagen BioRobot M48 workstation and the Qiagen MagAttract DNA Mini M48 Kit according to the manufacturer’s protocol.

### PCR systems and sequencing

Three PCR systems were used to type the adenovirus samples, targeting the genes of the viral DNA polymerase, the hypervariable sequence encoding the Arg-Gly-Asp-containing loop of the penton base, and a part of the hexon gene that encodes loop 1 (hypervariable region 1–6). The latter nested PCR was based on the work of Lu and Erdman [21]. The primers of the first two systems were designed based on publicly available conserved consensus sequences of HAdV-A to HAdV-G. The primer sequences are summarized in Table 1. The exact constitution and thermal profiles of the PCR reactions are detailed in S1 and S2 Tables.

**Table 1:** The primer sequences for the PCRs.

Targeted gene	Primer name		Primer sequence	Corresponding base pair ordinals of the HAdV-5 genome (AC_000008)	Product size (bp)	Reference
DNA polymerase	HAdV_pol_F		CTAYGSCATCTSGATCCARC	5,197–5,216	508–511	own design
	HAdV_pol_R		AGGTARGAARCGCATCAAAAA	5,687–5,707		
penton base	HAdV_pent_F		GTDGAYTTYACIBAIAGYCG	14,897–14,916	293–494	own design
	HAdV_pent_R		GMHARRWACCARCTICGRTA	15,362–15,382		
hexon	1^st^ round	AdhexF1	TICTTTGACATICGIGGIGTICTIGA	19,139–19,164	764–896	[21]
		AdhexR1	CTGTCIACIGCCTGRTTCCACA	19,998–20,019		
	2^nd^ round	AdhexF2	GGYCCYAGYTTYAARCCCTAYTC	19,169–19,191	714–855	
		AdhexR2	GGTTCTGTCICCCAGAGARTCIAGCA	19,959–19,984		

Without gel electrophoresis, the PCR products were sent to a commercial sequencing provider (Macrogen Europe) for Sanger sequencing. The sequences of the products were determined on both strands. As visualization of the PCR products was omitted, a PCR reaction was considered to be positive if the resulting assembled sequence contig measured 508–511 bp for the DNA polymerase-targeted PCR, 293–494 bp for the penton base-targeted PCR, and 714–855 bp for the hexon-targeted PCR. Otherwise, the reaction was considered to be negative. Mixed isolates were considered to be positive, but they were excluded from phylogenetic analysis.

Base calling and assembly of DNA polymerase and hexon sequences was performed using Tracetuner [22], and the assembly of penton base sequences was performed using Cap3 [23].

Recombinant genotypes are described using their penton base, hexon, and fiber sequences, and correct typing was difficult in some cases without having fiber-based data. Thus, we also performed a fiber gene-targeted PCR [20] for seven of the strains where the other three regions did not allow us to type the strain unambiguously (UmU053, UmU061, UmU082, UmU219, UmU234, UmU262, and UmU271).

### Rough typing of strains based on partial sequences

The strains were typed molecularly based on the derived amino acid sequence of all three (or four) sequence stretches. For all stretches amplified, the most closely related (i.e. with highest sequence identity, in other words, the least distant) HAdV reference strain was determined using the pairwise-alignment-based Sequence Demarcation Tool (SDT) v1.2 [24].

Each reference strain (also called prototype) represented a different HAdV type. Up to serotype ordinal 41 (HAdV-41), the strains recommended by Horwitz [25] were used. For types HAdV-53 to HAdV-84, we used the strains recommended by the Human Adenovirus Working Group (http://hadvwg.gmu.edu/). For types HAdV-42 to HAdV-52, we chose a strain with the complete genome sequence available.

As DNA polymerase and penton base sequences were not sufficiently variable (i.e. more reference strains shared identical or very closely related sequence stretches), a type assessment was reached, in most cases based on the hexon result. If this raised the possibility that the strain represented a previously described recombinant type, the sequence identity values of the DNA polymerase and penton base were also evaluated carefully (see example below). In such a case, we investigated whether these differed from the hexon result, which would suggest a final type assessment as a recombinant genotype. For example, the hexon amplimer of strain UmU044 showed the highest sequence identity with that of HAdV-64 (S3 Table), and the second highest with that of HAdV-19 (98.5%). Its DNA polymerase amplimer was identical to those of HAdV-37, -60, and -64, and its penton base amplimer was identical to those of HAdV-22, -42, -59, and -64. As HAdV-64 is a recombinant of HAdV-22, -19, and -37 (penton base, hexon, and fiber, respectively) [26], UmU044 was typed as HAdV-64.

To be able to identify divergent HAdV strains, we determined the minimum level of divergence between two distinct, known serotypes. That is, the two most closely related non-recombinant HAdV reference strains (serotypes) were determined based on all three sequence stretches corresponding to the PCR products. Primers were omitted from the analysis. The corresponding base pair ordinals of the HAdV-5 genome (AC_000008) were 5217–5686 for the DNA polymerase, 14917–15361 for the penton base, and 19192–19958 for the hexon genes.

### Complete genome sequences

Based on the typing results, four divergent strains (UmU010, UmU193, UmU225, and UmU253) were identified. Furthermore, UmU018 had a potentially novel genome composition that had not been seen previously. For further characterization, these five clinical isolates were propagated on human alveolar epithelial cells (A549). Intracellular viral DNA was purified from infected cells using the protocol of Kajon and Erdman [27]. The genomes of UmU010, UmU193, and UmU225 were sequenced using Ion Torrent next-generation sequencing at the Uppsala Genome Center of the National Genomics Infrastructure (SciLifeLab; Uppsala, Sweden). We did not succeed in producing the required amount of DNA of UmU018 and UmU253 in the first round, and for the quality required, UmU193 also required supplementation with additional sequence information, so these three strains were sequenced in the next round using Illumina HiSeq2500 technology at GATC Biotech (Konstanz, Germany). The resulting reads were normalized to a 60-times coverage using BBNorm from the BBTools suite. The normalized reads were assembled *de novo* using Mira version 4.9.5_2 [28], and the original sequence reads were mapped to the resulting consensus sequences using the Geneious mapper at the highest sensitivity with five iterations in Geneious 9.1.8 [29]. The new consensus sequences were annotated based on HAdV reference strain genome annotations, using the Annotate & Predict function of Geneious. The annotations were checked manually and edited.

### Accession numbers

The sequences from the virus strains were deposited in GenBank (NCBI; accession numbers: KX868282–KX868551).

### Phylogenetic analysis of complete genomes

To investigate their phylogenetic relationship, the completely sequenced strains were analyzed using both multiple-alignment-based phylogenetic tree inference and pairwise-alignment-based sequence identity calculation. Both types of analyses were based on complete genome sequences and derived amino acid sequences of the entire hexon, penton base, and DNA polymerase; and also hexon loop 1 (delimited according to Yuan et al. [30]) and the fiber knob. As UmU253 had two fibers, both fiber knobs were analyzed.

For phylogenetic tree inference, multiple alignments were conducted using MAFFT [31], and phylogenetic calculations were performed using RAxML 8.2.10 [32] based on alignments edited in Gblocks 0.91b [33]. Evolutionary model selection for the complete genome sequence alignment was performed using MEGA 7 [34], and using RAxML for the protein alignments. The robustness of the trees was determined with a non-parametric bootstrap calculation using 1,024 repeats. To accelerate phylogenetic analyses, the calculations were run in parallel on 32 processor cores. RAxML always calculates integer multiples of the cores used, and uses the smallest value above the desired one as the number of replicates. In this case, 1,000 replicates were targeted, so 1,024 (32*32) were used. Phylogenetic trees were visualized using MEGA 7 [34], trees were rooted on the midpoint, and bootstrap values are given as percentages if they reached 75%.

Pairwise-alignment-based sequence identity calculations were conducted using the MAFFT alignment algorithm in SDT [24, 31]. The most closely related HAdV type (reference strain) was determined for each completely sequenced strain, based on all sequence stretches analyzed. Next, those non-recombinant *sero*types were indicated which showed the highest level of sequence identity with each other. It is important to stress the word “*sero*type” here. By determining the minimum divergence between two distinct non-recombinant serotypes, we could evaluate the level of divergence of the completely sequenced strains.

Recombination events in strain UmU018 were analyzed further using SimPlot 3.5.1 [35] based on the complete genome alignment and subslices corresponding to the penton base gene, the hexon gene, and the E3 region.

## Results

### Efficiency of PCR amplification

Using the PCR systems targeting the hexon gene, the polymerase gene, and the penton base gene, we found that the virus strains gave 99.3%, 94.7%, and 77.9% positivity, respectively, and that 5.4%, 6.4%, and 2.3% of the sequences analyzed were mixtures. Such mixture reads were considered to be positive but we excluded them from the subsequent phylogenetic analysis.

### Rough typing results based on partial sequences

The results of typing of the strains are summarized in S3 Table. Analysis of part of the DNA polymerase gene revealed that 71.5% of the strains that gave DNA polymerase sequences belonged to species HAdV-C, but almost all other human adenovirus species were found as well, with the exception of species G. In the samples, 18 different HAdV types were identified; the most common being HAdV-2 (38.1%) and HAdV-1 (22.8%). We conducted preliminary analyses comparing the calculated sequence identities (S3 Table) to the ones measured between different HAdV types (data not shown). These indicated that four strains (UmU010, UmU193, UmU225, and UmU253) diverged significantly from the closest HAdV reference strain, so the four complete genomes were sequenced.

At least two of the three PCR analyses provided sequence information for 251 strains, and seven of these were found to be recombinants. Furthermore, as UmU114 was typed as HAdV-60 based on the hexon gene only, this strain was also classified as a recombinant (S3 Table). Most of these recombinants were already accepted HAdV types, e.g. HAdV-64 (n = 3).

In the beginning of this study, recombinant strains were identified erroneously, based on the DNA polymerase, penton base, and hexon sequences only. For example, UmU053 was identified first as HAdV-66 (a recombinant genotype), as its penton base showed highest amino acid identity to HAdV-7, and its hexon to HAdV-66. But the genomic composition of HAdV-66 is an HAdV-7 penton base, an HAdV-7 hexon, and an HAdV-3 fiber. So, to check this result, the fiber gene sequence of UmU053 (and also six other strains: UmU061, UmU082, UmU219, UmU234, UmU262, and UmU271) was also determined, which contradicted the previous typing result (in the other cases too). These strains were originally typed as recombinant HAdV-66 or HAdV-68 based on their hexon sequences, but the HAdV-3 or HAdV-7 fiber sequences showed that these strains were common HAdV-3 (UmU219, UmU234, UmU262, and UmU271) or HAdV-7 (UmU053, UmU061, and UmU082). Without fiber data, such strains could not be typed accurately, so two types are given as the final type identified (S3 Table), e.g. HAdV-7 or HAdV-66 for UmU062.

UmU018 had a new combination of adenovirus genomes. The hexon loop 1 of this strain showed high identity (99.2%) to HAdV-25, its penton base showed high identity to HAdV-9, -10, -26, and -56 (99.0%), and its DNA polymerase showed high identity to 37 different HAdV-D types, including HAdV-9, -10, -25, and -26 (98.7–99.4%). This strain was chosen for complete genome sequencing.

### Complete genomes and their phylogenetic analysis

The read-coverage reports and basic attributes of the genomes are summarized in S4 Table.

**Fig 1:**
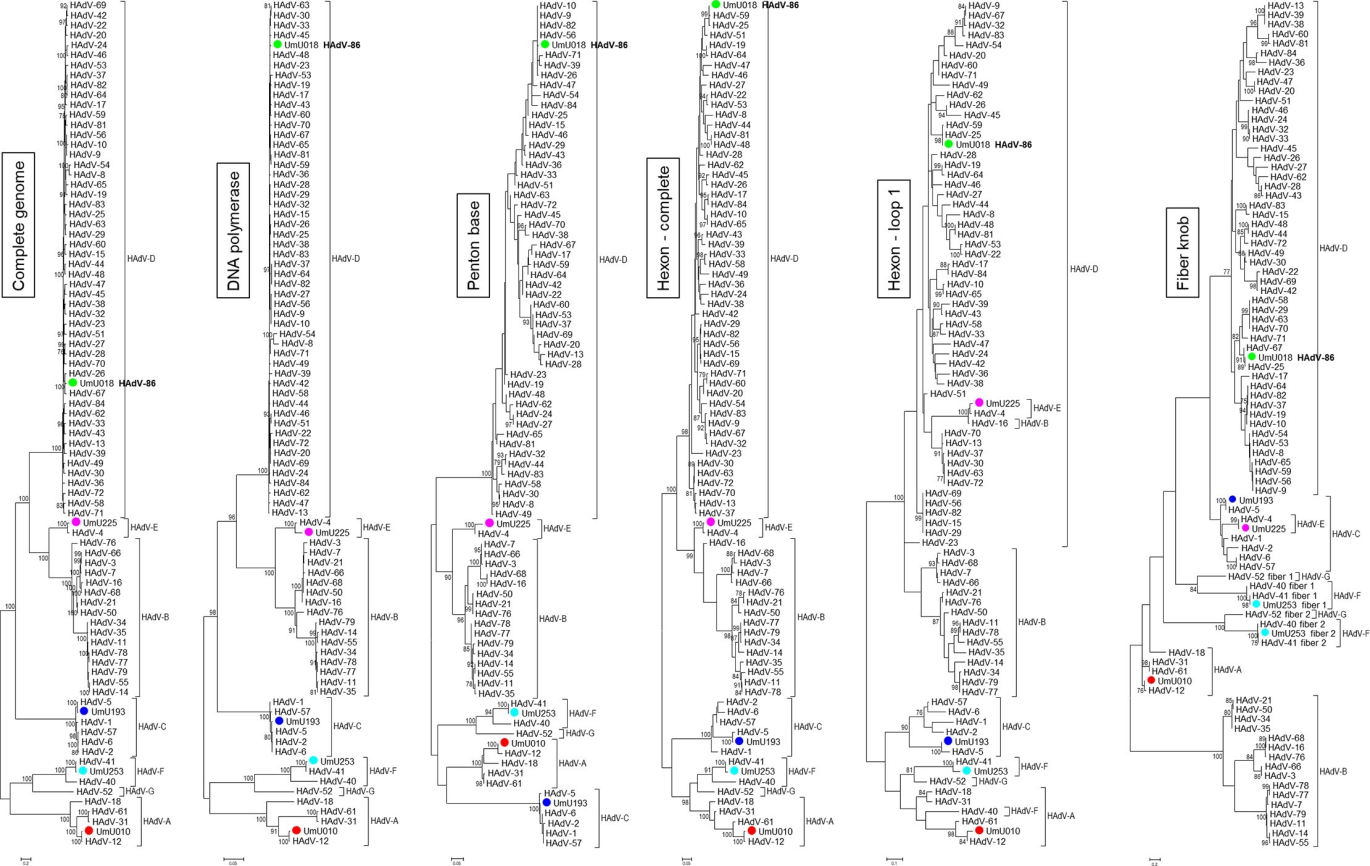
Phylogenetic analysis of the five completely sequenced human adenovirus strains. The complete genome analysis was based on nucleotide sequences; all other analyses were based on derived amino acid sequences.

**Fig 2:**
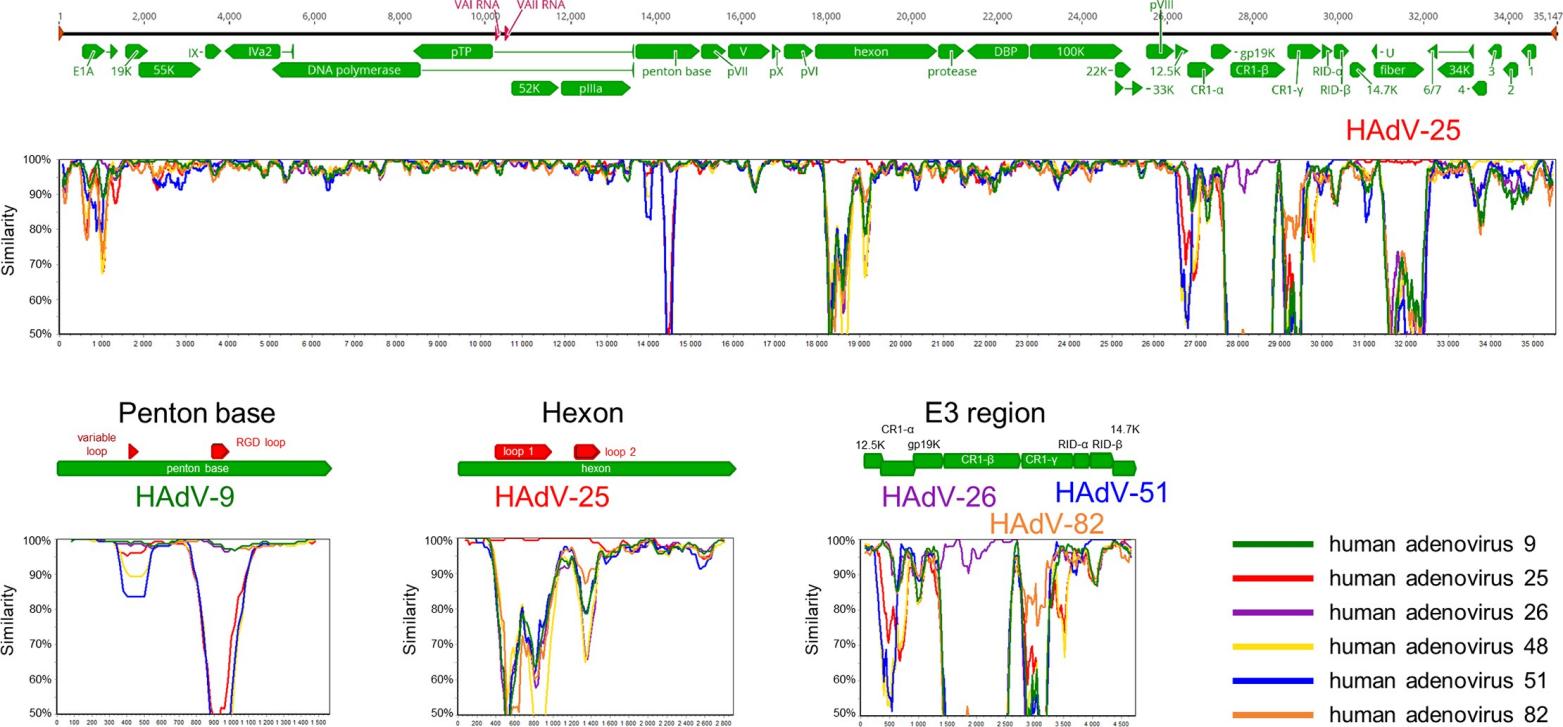
Genomic layout and recombination analysis of human adenovirus type 86 (HAdV-86), strain UmU018. Green arrows in the genome map represent protein coding sequences, red arrows represent virus-associated RNAs, and brown arrows represent the inverted terminal repeats. Human adenovirus types showing the highest sequence identity in characteristic domains or coding sequences are shown abbreviated (e.g. HAdV-9 means human adenovirus 9).

All phylogenetic tree inferences (Fig 1) and sequence identity analyses (Table 2) confirmed that UmU010, UmU193, UmU225, and UmU253 were most closely related to HAdV-12, HAdV-5, HAdV-4, and HAdV-41, respectively. The only two exceptions were the DNA polymerase and penton base analyses of UmU193, where HAdV-6 was found to be most closely related to this strain, though HAdV-5 also gave very high degrees (percentages) of identity. The recombinant strain UmU018 had a DNA polymerase that was most similar to that of HAdV-48, -58, and -65, whereas its penton base showed high amino acid sequence identity to HAdV-9, -10, -56, and -82. To find the most closely related HAdV type, the stretch of penton base was also analyzed at the nucleic acid level, and showed the highest level of sequence identity to HAdV-9 (99.17%). The hexon and fiber proteins of UmU018 were most similar to those of HAdV-25 (Figs 1 and 2, and Table 2). Furthermore, SimPlot analysis revealed that its E3 genomic region was a recombinant of HAdV-26, -51, and -82 (Fig 2); and the derived amino acid sequences of the DNA polymerase, core protein V, and the 100 K protein showed high levels of sequence identity to several species D types (Table 3).

**Table 2:** Sequence identity-based typing results for the five sequenced strains.

Compared stretch	HAdV serotypes, closest related to each other, and their sequence identity		Closest related HAdV type and its sequence identity to the investigated strain										
			UmU010		UmU018		UmU193			UmU225		UmU253	
									Comp. to HAdV-5				
Compl. genome (NA)	11–35	98.28%	12	98.56%	67	96.26%	5	99.09%		4	95.69%	41	99.16%
DNA polymerase (AA)	9–10	100.00%	12	99.49%	48*	99.63%	6	99.58%	99.16%	4	96.81%	41	99.75%
Penton base (AA)	9–10	100.00%	12	98.59%	9^†^	99.61%	6	99.47%	99.30%	4	97.20%	41	100.00%
Hexon—complete (AA)	13–30	98.08%	12	98.37%	25	99.48%	5	98.74%		4	98.61%	41	98.27%
Hexon—loop 1 (AA)	13–30	95.24%	12	93.10%	25	98.98%	5	96.60%		4	95.24%	41	93.37%
Fiber knob (AA)	13–38	100.00%	12	96.55%	25	99.44%	5	99.44%		4	98.85%	F1: 41 F1 F2: 41 F2	F1: 98.68% F2: 100.00%

The HAdV serotype (reference strain) most closely related to the completely sequenced human adenovirus strains was determined. This comparison was conducted on six sequence stretches: complete genome nucleic acid (row 2), derived amino acid sequences of the complete DNA polymerase, penton base, and hexon (rows 3–5), and also the derived amino acid sequences of hexon loop 1, and the fiber knob (rows 6–7). As strain UmU253 had two fibers, both fiber knobs were analyzed. In the two cases where UmU193 showed highest sequence identity to HAdV-6, the HAdV-5 percentages are also indicated. The most closely related non-recombinant HAdV serotypes (reference strains) were also determined based on all sequence stretches analyzed: here meaning the serotypes most closely related to each other (coloumn 2). Human adenovirus types are represented by their type number only. Abbreviations: AA, amino acid; comp., compared; compl., complete; F1, fiber 1; F2, fiber 2; HAdV, human adenovirus; NA, nucleic acid.

* The same sequence identity was measured compared to the derived DNA polymerase amino acid sequence of HAdV-48, -58, and -65.

^†^ The same sequence identity was measured compared to the derived penton base amino acid sequence of HAdV-9, -10, -56, and -82.

**Table 3:** Closest BlastP hits (04/16/2018) of three conserved, less variable protein-coding sequences originating from human adenovirus 86 (HAdV-86, strain UmU018).

Coding sequences	BlastP hits	
	Identity	HAdV types
DNA polymerase	99.6%	48, 58, 65
Core protein V	98.2–99.1%	17, 24, 32, P38H32F27
100 K	99.3–99.5%	10, 44, 56, 72, P67H9F15

The five sequenced strains were evaluated by the Human Adenovirus Working Group as possible new HAdV type candidates, and UmU018 (complete strain designation: Adenovirus D human/SWE/UmU018/1978/86[P9H25F25]) was approved as HAdV genotype 86.

## Discussion

In this era of quick, high-throughput, and cheap sequencing methods, one might easily question the rationale behind using a PCR-based typing system for HAdVs, especially when we consider the very limited amount of information gained by using it. Even so, at least at the present time, no real practical alternatives are available for the screening of hundreds of virus strains. Target enrichment and genome sequencing methods do improve, become cheaper, and certainly provide abundant information about the strain under examination: this is undoubtedly the future of virus typing. However, available target enrichment methods require the PCR amplification of the enriched fragment pool [36], so they do not provide any real advantages over the method detailed here. We also considered propagating all strains to obtain the amounts of DNA required for complete genome sequencing. However, this would have been far too expensive and time consuming. Thus, we used this PCR-based screening method instead.

A quick HAdV typing system would benefit researchers, e.g. epidemiologists, as at least a partial genomic composition of an HAdV strain could be determined. Although there are several typing systems available, many HAdV typing systems are limited: (1) some systems are not generic [37, 38]; (2) diverse HAdV types may be detected using multiple PCRs [3, 4, 20, 39, 40]; (3) the antigenic determinant loops may be excluded [5, 20, 41]; (4) even if the loops are included, the product may be too long for convenient sequence analysis [42, 43]; (5) recombinant strains can only be detected by a very complex algorithm [44, 45]; or (6) the system may target only one gene, excluding the possibility of recombinant detection [5, 21, 38, 40–42, 46]. Our multigene HAdV typing system provides a solution to the six issues mentioned above by providing sequence information from three characteristic regions of the HAdV genome.

Nowadays, if information about more than a single gene is needed, this is usually achieved by sequencing of the complete genome [47, 48]. Although this approach provides abundant information about the strain being examined, it is still more time consuming (requiring virus isolation) and more expensive than a PCR-based multigene typing system. Isolates were analyzed in our study also, but DNA derived from clinical material is often used in many PCR-based diagnostic methods. The hexon gene-targeted PCR, for example, was tested in the original publication and also later using clinical specimens [21, 49]. Thus, it is likely that our typing system would give at least partial positivity using clinical material, and this is its main advantage over contemporary complete genome typing methods, as virus isolation and propagation can be time consuming.

As the hexon gene-targeted PCR had been validated previously using a panel of 51 HAdV types [21], we did not conduct such an analysis. In the present study, the penton base-targeted PCR had the lowest positivity rate (77.9%), but this result might only have been a consequence of the limited PCR sensitivity, possibly caused by highly degenerate primers. The possibility of selective specificity was excluded, as every HAdV species was detectable using it. Using the typing system, we detected 6 HAdV species among the samples; HAdV-G was the exception. The hexon primers had been designed based on HAdV-A to HAdV-F sequences only [21]. However, as the penton base gene- and DNA polymerase gene-targeted PCRs were designed based on reference sequences from HAdV-A to HAdV-G, it is quite probable that all HAdV-G strains would yield a positive result using these. Thus, our generic HAdV multigene typing system is capable of providing sequence information from all HAdV species on (1) the hexon loop 1, (2) the hypervariable loop of the penton base, and (3) the DNA polymerase―the first two of which are antigenic determinants [14] and the latter is the main species determinant [1].

Besides sensitivity, another important factor for typing systems is their resolution capacity. Being the major antigenic determinants, hexon loops 1 and 2 are often used in molecular typing of HAdV strains [3, 4, 42, 46, 50] and provide a type-specific sequence for the non-recombinant HAdV serotypes. This was confirmed in the study providing the hexon gene-targeted primers, where 51 reference adenovirus strains (formerly demarcated using serotyping) were analyzed [21]. The same homologous sequence stretch, originating from the same and newer reference strains, was analyzed *in silico* in our study (now representing 86 HAdV genotypes), providing the same result: non-recombinant HAdV strains can be typed based on hexon loop 1 amino acid sequences; that is, non-recombinant HAdV types can be determined using this sequence stretch. Combining the hexon data with penton base and fiber results, we could achieve a preliminary typing of any HAdV strain, even recombinant ones, as all accepted HAdV genotypes have a unique combination of penton base, hexon, and fiber genes [19, 51]. Unfortunately, fiber data could not be obtained with our approach, and some closely related types share identical or very similar penton base stretches, as described already by Ismail et al. [52]. This makes distinction impossible for the following ten pairs of 19 genotypes using only DNA polymerase, penton base, and hexon information: HAdV-3 and -68, -7 and -66, -11 and -55, -11 and -78, -15 and -29, -20 and -60, -21 and -76, -30 and -63, -56 and -82, and finally -77 and -79. All other recombinant or recently described HAdV types (HAdV-16, -53, -54, -57–59, -61, -62, -64, -65, -67, -69–75, -80, -81, and -83–86) can be distinguished from the parental types and any other types using the combination of hexon and penton base amino acid sequences.

In conclusion, this typing system is not entirely optimal to completely describe or type an HAdV strain. For definite typing, complete genome information is required [19, 51], as homologous recombination is one of the major driving forces in HAdV evolution [6, 52–55]. Alternatively, if not the complete genome, at least fiber information is crucial in addition to the hexon and penton base sequences [19]. However, the system developed is adequate for preliminary typing in some cases. In addition, a new recombinant HAdV strain was found using this typing system, as discussed in detail below; furthermore, divergent strains of established types were also detected in various HAdV species. Users must weigh the advantages and disadvantages of the methods available, as well as the time available, the budget, and the number of samples.

Of the strains analyzed, we observed a very high proportion of HAdV-C strains, but almost all other human adenovirus species (HAdV-A to HAdV-F) were also found. A similar predominance of HAdV-C strains was observed by Sriwanna et al. [56]. Al Qurashi et al. also detected strains belonging to six HAdV species, and a similar high proportion of HAdV-C, but there was a higher proportion of species HAdV-A types as compared to our results [57]. In other studies, more diverse strain distribution or a higher proportion of HAdV-B was observed [21, 58, 59]. The diverse nature of the clinical samples investigated in this study might explain this result: our study was not limited to respiratory samples, for example.

Mixture sequences originate from co-infections, which happens often [45]. Based on hexon sequences, we found that 5.4% of the isolates that gave a positive PCR reaction contained at least two different HAdV types, which is similar to the 3.5% observed by McCarthy et al. [45] but lower than the 18.2% observed by Metzgar et al. [44]. In eight cases out of the 15, the DNA polymerase sequence could be interpreted, showing that the co-infecting strains belonged to the same species.

Every strain that was typed as HAdV-5 based on the hexon loop 1 sequence had a penton base stretch closest to that of HAdV-1, and 90 of the 99 strains typed as HAdV-2 based on the hexon loop 1 sequence also had a DNA polymerase stretch closest to that of HAdV-1. As there is a mere two-amino-acid difference between the penton base loops of HAdV-1 and HAdV-5 and a one-amino-acid difference between the HAdV-1 and HAdV-2 DNA polymerase stretch analyzed, these strains were not regarded as recombinants. As previously observed in penton base sequences [52], DNA polymerase sequences might also be enough only for species classification.

In the strains examined, we also tried to identify possible new HAdV candidate types. We wanted to enable the discovery of possible new types based on both traditional and contemporary techniques, and this could be achieved by our multigene molecular typing system. We aimed to identify (1) new, divergent, distance-based types, showing low sequence identity to the closest related reference strain―earlier this would have been a new serotype, and (2) new and unpublished recombinants―new genotypes.

Combining the three different typing results, we discovered one possible new recombinant, UmU018, with a previously unpublished combination of HAdV genomes. This strain has been approved by the Human Adenovirus Working Group to represent a novel genotype: HAdV-86. Similar to previous findings in the case of HAdV-84 [6], HAdV-86 has a general HAdV-D backbone: conserved core proteins show very high sequence identity to several different closely related HAdV-D types. The surface proteins with antigenic determinants showed a close relationship to those of HAdV-9 and -25. Perhaps the exact direction of evolution cannot be determined based on these similarities, e.g. the penton base loops may have originated from HAdV-9, or HAdV-9 may have inherited these from HAdV-86, or both of these types inherited them from a common, but unknown ancestor. Homologous recombination has a crucial role in HAdV evolution [6, 52–55], as it provides the progeny strain with a new set of major antigenic determinants. This enables a quick antigenic shift in a possibly neutralizing environment, or the use of novel receptors. The E3 region is also a hotspot for recombinations in the HAdV genome [60]. Most of the HAdV-86 strain’s E3 region is closely related to that of HAdV-26, but the CR1-gamma represents a novel proteotype. The amino acid sequence of the CR1-gamma protein shows only 77.9–81.5% identity to that of HAdV-22, -37, or -82, and the threshold for a novel proteotype was set at 10% divergence [60]. UmU018 was isolated in 1978; the only information available suggests that the patient was investigated for infection by tropical pathogens.

The other four sequenced strains cannot be recognized as new HAdV types, as the imputed serology used does not always correlate strongly with serotyping, and the minor sequence differences observed might not represent sufficient differences in a conventional serum neutralization assay. Also, recognizing these strains as new types could cause considerable confusion in the field. As a member of the Human Adenovirus Working Group pointed out, for example, UmU225 is almost identical to field strains NHRC 3 and NHRC 42606 (complete genome nucleic acid identity: 99.85% and 99.96%, respectively), both of which were typed as HAdV-4, and historically within this serotype as genome type HAdV-4a [61]. A virtual restriction endonuclease cleavage analysis of the UmU225 genome also supported classification of the strain as HAdV-4a (data not shown). Although this genome type is divergent from the reference strain (RI-67, genome type: HAdV-4p, “p” meaning prototype or reference strain), it can still be identified as serotype 4 (HAdV-4) by neutralization test [62].

A straightforward typing system might help improve epidemiological investigations. Here we have presented a rapid, generic, multigene typing system for human adenoviruses that can characterize three main deterministic regions of HAdV strains and thus distinguish the non-recombinant HAdV types, and most recombinant HAdV types also. Using this system, we analyzed 281 HAdV clinical isolates, and could detect not only divergent strains of established types, but also a new recombinant HAdV strain with a previously unpublished combination of HAdV genomes. This strain was approved by the Human Adenovirus Working Group to represent a novel genotype: HAdV-86.

## Supporting information

S1 Table Constitution of the polymerase chain reactions used. (DOCX)

S2 Table Thermal profiles of the polymerase chain reactions used. (DOCX)

S3 Table Rough typing results of Swedish human adenovirus clinical isolates based on partial amino acid sequences. (XLSX)

S4 Table Sequence read coverage and basic attributes of the completely sequenced genomes. (DOCX)
