# Supplementary material for: A multigene typing system for human adenoviruses reveals a new genotype in a collection of Swedish clinical isolates
Source: PLoS One. 2018 Dec 14;13(12):e0209038. doi: 10.1371/journal.pone.0209038 (PMC6294355; doi:10.1371/journal.pone.0209038)
Supplement: S1 Table — (DOCX) [file pone.0209038.s001.docx]

**S1 Table.** Constitution of the polymerase chain reactions used

|  | DNA polymerase-targeted | Penton base-targeted | Hexon-targeted (both rounds) |
| --- | --- | --- | --- |
| Ultrapure water | 18.6 | 17.6 | 21 |
| OneTaq Hot Start Quick-Load 2X Master Mix with Standard Buffer (New England Biolabs) | 25 | 25 | 25 |
| MgCl_2_ (25 mM) | 4.4 | 4.4 | 0 |
| Primer 1^a^ | 0.5 | 0.5 | 1 |
| Primer 2^a^ | 0.5 | 0.5 | 1 |
| DNA sample | 1 | 2 | 2 |
| **End volume** | **50** | **50** | **50** |

All volumes are in microliters.

^a^Primer concentration for the DNA polymerase- and the penton base-targeted PCRs: 100 µM. Primer concentration for the hexon-targeted PCRs: 50 µM.
