# Supplementary material for: A multigene typing system for human adenoviruses reveals a new genotype in a collection of Swedish clinical isolates
Source: PLoS One. 2018 Dec 14;13(12):e0209038. doi: 10.1371/journal.pone.0209038 (PMC6294355; doi:10.1371/journal.pone.0209038)
Supplement: S2 Table — (DOCX) [file pone.0209038.s002.docx]

**S2 Table.** Thermal profiles of the polymerase chain reactions used

| DNA polymerase-targeted | | | Penton base-targeted | | | Hexon-targeted (both rounds) | | |
| --- | --- | --- | --- | --- | --- | --- | --- | --- |
| Temperature (°C) | Duration (s) | No. of cycles | Temperature (°C) | Duration (s) | No. of cycles | Temperature (°C) | Duration (sec) | No. of cycles |
| 95 | 600 | 1 | 95 | 600 | 1 | 94 | 120 | 1 |
| 95 | 60 | 40 | 95 | 60 | 40 | 94 | 60 | 35 |
| 40 | 30 |  | 40 | 60 |  | 45 | 60 |  |
| 72 | 40 |  | 72 | 60 |  | 72 | 120 |  |
| 72 | 360 | 1 | 72 | 360 | 1 | 72 | 300 | 1 |
| 4 | hold | 1 | 4 | hold | 1 | 4 | hold | 1 |
