# Supplementary material for: A multigene typing system for human adenoviruses reveals a new genotype in a collection of Swedish clinical isolates
Source: PLoS One. 2018 Dec 14;13(12):e0209038. doi: 10.1371/journal.pone.0209038 (PMC6294355; doi:10.1371/journal.pone.0209038)
Supplement: S4 Table — (DOCX) [file pone.0209038.s004.docx]

**S4 Table.** Sequence read coverage and basic attributes of the completely sequenced genomes

| Strain name | UmU010 | UmU018 | UmU193 | UmU225 | UmU253 |
| --- | --- | --- | --- | --- | --- |
| Complete strain designation | Adenovirus A human/SWE/UmU010/  1978/12[P12H12F12] | Adenovirus D human/SWE/UmU018/  1978/86[P9H25F25] | Adenovirus C human/SWE/UmU193/  2006/5[P5H5F5] | Adenovirus E human/SWE/UmU225/  2007/4[P4H4F4] | Adenovirus F human/SWE/UmU253/  2008/41[P41H41F41] |
| Minimum coverage | 38 | 18,681 | 1,903 | 7 | 24,745 |
| Mean coverage | 1,252.0 | 82,482.2 | 27,725.7 | 908.1 | 56,194.1 |
| Standard deviation of the coverage | 277.5 | 13,792.7 | 7,219.2 | 241.8 | 23,149.9 |
| Average of base quality sums | 32,479.2 | 2,719,498.1 | 906,461.4 | 24,889.6 | 1,703,684.4 |
| Genome length (bp) | 33,676 | 35,147 | 35,918 | 35,969 | 34,184 |
| G+C content (%) | 46.7 | 57.0 | 55.2 | 56.3 | 51.0 |
| No. of coding sequences | 35 | 37 | 36 | 37 | 34 |
| Inverted terminal repeat length (bp) | 164 | 150 | 103 | 209 | 156 |
| Type classification | human adenovirus 12 | human adenovirus 86 | human adenovirus 5 | human adenovirus 4 | human adenovirus 41 |
| Species classification | *Human mastadenovirus A* | *Human mastadenovirus D* | *Human mastadenovirus C* | *Human mastadenovirus E* | *Human mastadenovirus F* |
